# The Effects of L1 English Constraints on the Acquisition of the L2 Spanish Alveopalatal Nasal

**DOI:** 10.3389/fpsyg.2021.640354

**Published:** 2021-02-15

**Authors:** Sara Stefanich, Jennifer Cabrelli

**Affiliations:** ^1^Department of Spanish and Portuguese, Northwestern University, Evanston, IL, United States; ^2^Department of Hispanic and Italian Studies, University of Illinois at Chicago, Chicago, IL, United States

**Keywords:** second language acquisition, phonology, phonetics, spanish, english, nasals

## Abstract

This study examines whether L1 English/L2 Spanish learners at different proficiency levels acquire a novel L2 phoneme, the Spanish palatal nasal /ɲ/. While alveolar /n/ is part of the Spanish and English inventories, /ɲ/, which consists of a tautosyllabic palatal nasal+glide element, is not. This crosslinguistic disparity presents potential difficulty for L1 English speakers due to L1 segmental and phonotactic constraints; the closest English approximation is the heterosyllabic sequence /nj/ (e.g., “canyon” /kænjn/ ['k^h^æn.jn], cf. Spanish *cañón* “canyon” /kaɲon/ [ka.'ɲon]). With these crosslinguistic differences in mind, we ask: (1a) Do L1 English learners of L2 Spanish produce acoustically distinct Spanish /n/ and /ɲ/ and (1b) Does the distinction of /n/ and /ɲ/ vary by proficiency? In the case that learners distinguish /n/ and /ɲ/, the second question investigates the acoustic quality of /ɲ/ to determine (2a) if learners' L2 representation patterns with that of an L1 Spanish representation or if learners rely on an L1 representation (here, English /nj/) and (2b) if the acoustic quality of L2 Spanish /ɲ/ varies as a function of proficiency. Beginner (*n* = 9) and advanced (*n* = 8) L1 English/L2 Spanish speakers and a comparison group of 10 L1 Spanish/L2 English speakers completed delayed repetition tasks in which disyllabic nonce words were produced in a carrier phrase. English critical items contained an intervocalic heterosyllabic /nj/ sequence (e.g., ['p^h^an.jə]); Spanish critical items consisted of items with either intervocalic onset /ɲ/ (e.g., ['xa.ɲa]) or /n/ ['xa.na]. We measured duration and formant contours of the following vocalic portion as acoustic indices of the /n/~/ɲ/ and /ɲ/ ~/nj/ distinctions. Results show that, while L2 Spanish learners produce an acoustically distinct /n/ ~ /ɲ/ contrast even at a low level of proficiency, the beginners produce an intermediate /ɲ/ that falls acoustically between their English /nj/ and the L1 Spanish /ɲ/ while the advanced learners' Spanish /ɲ/ and English /nj/ appear to be in the process of equivalence classification. We discuss these outcomes as they relate to the robustness of L1 phonological constraints in late L2 acquisition coupled with the role of perceptual cues, functional load, and questions of intelligibility.

## Introduction

A lasting question that has occupied a central role in the study of second language (L2) phonology across several decades asks which factors modulate the acquisition of L2 contrastive sounds that are not part of the first language (L1) grammar. A look at the collective body of research reveals that, while there is robust evidence that novel L2 sounds are acquirable (see e.g., Broselow and Kang, 2013, for a review), it is clear that not all sounds are equal when it comes to their acquirability. A sound's degree of difficulty can depend on a number of variables, which span the existence or absence of a phonologically similar L1 sound, functional load of the L2 sound, markedness, articulatory complexity, and language-specific constraints (featural and suprasegmental alike), among other factors.

In the present study, we examine L1 American English speakers' acquisition of the alveopalatal nasal /ɲ/ in L2 Spanish. This sound is a challenge for L1 American English speakers for a number of reasons. First, this is a scenario in which the L2 sound does not exist in the L1. Second, it is the least frequent phoneme in the Spanish inventory (Melgar de González, 1976) and has low functional load in Spanish. Third, L1 segmental and phonotactic constraints complicate the L2 learning task: American English does not permit complex palatal segments and the closest approximation in the English inventory is the sequence /nj/ (e.g., “canyon” /kænjn/ ['k^h^æn.jn], which is derived from Spanish *cañón* “canyon” /kaɲon/ [ka.'ɲon]), which is restricted to heterosyllabic position. With these crosslinguistic differences in mind, to converge on the L2 Spanish target, the learner's grammar must come to allow a single alveopalatal nasal segment. The question, then, that follows, is whether these constraints can be overcome in the L2. While there are no L2 Spanish /ɲ/ acoustic or perception data to inform our predictions for the current study^1^, we can look to a body of work that has examined L1 English speakers' acquisition of L2 Russian palatalized consonants to inform predictions for L2 Spanish learners. Specifically, L2 Russian learners have been reported to persistently rely on L1 /Cj/ sequences in both perception and production (e.g., Diehm, 1998), which leads to the prediction that L2 Spanish learners will pattern similarly to L2 Russian speakers and fail to reliably produce an alveopalatal nasal segment. In this study, we report acoustic data from a delayed repetition task completed in English and Spanish by L1 English learners of L2 Spanish at beginner and advanced levels of proficiency and in Spanish by a baseline comparison group of L1 Spanish/L2 English speakers. While L2 Spanish learners produce an acoustically distinct /n/ ~ /ɲ/ contrast even at a low level of proficiency, the beginners produce an intermediate /ɲ/ that falls acoustically between their English /nj/ and the L1 Spanish /ɲ/ while the advanced learners' Spanish /ɲ/ and English /nj/ appear to be in the process of equivalence classification. We discuss these outcomes as they relate to the robustness of L1 phonological constraints in late L2 acquisition coupled with the role of functional load and questions of intelligibility. In the remainder of this section, we outline the phonetic and phonological properties of the nasal segments and sequences in English and Spanish and the L2 learning task, followed by a brief overview of the L2 research that informs our research questions and predictions and the questions and predictions themselves.

### Nasal Consonants in English and Spanish

Spanish and English each have three nasal phonemes that contrast by place of articulation; however, while the Spanish nasal inventory (/m/ /n/ /ɲ/) includes an alveopalatal nasal that contrasts with the other nasals in word-medial onset position^2^, the English inventory (/m/ /n/ /η/, the latter of which is limited to coda position) does not.

**Table:** 

1.	/m/	*cama*	/'ka**m**a/	‘bed’
	/n/	*cana*	/'ka**n**a/	‘gray hair’
	/ɲ/	*caña*	/'ka**ɲ**a/	‘cane’

According to Martínez Celdrán and Fernández Planas (2007), the alveopalatal /ɲ/ is comprised of an alveolar nasal segment and a “partial” glide element. This glide element is posited to be phonologically associated with the nasal segment (e.g., Colina, 2009; Bongiovanni, 2019). An alveopalatal onset is illicit in American English due to two phonological constraints. First, as noted, American English does not allow palatal consonants with complex (simultaneous or sequential) points of articulation; instead, consonantal palatalization is realized non-contrastively as a sequence of distinct consonantal and glide segments (e.g., “music” [mju:zik], cf. [m^j^u:zik]) (e.g., Antonova, 1988, cited in Diehm, 1998). As a result, /nj/ will be the closest American English approximation to Spanish /ɲ/. Second, American English^3^ /nj/ cannot occupy onset position due to a ban on onset clusters that consist of a coronal segment and /j/ (see Kulikov, 2011). Rather, /nj/ is limited to a heterosyllabic context in which /n/ occupies a syllable coda and /j/ is phonologically associated with the following syllable onset (consider, for example, “canyon” /kænjn/ ['k^h^æn.jn], which is derived from Spanish *cañón* /kaɲon/ [ka.'ɲon]). Together, these L1 constraints yield a learning task in which the grammar must come to allow a single nasal segment alveolar and palatal places of articulation in syllable onset position.

**Figure 1:**
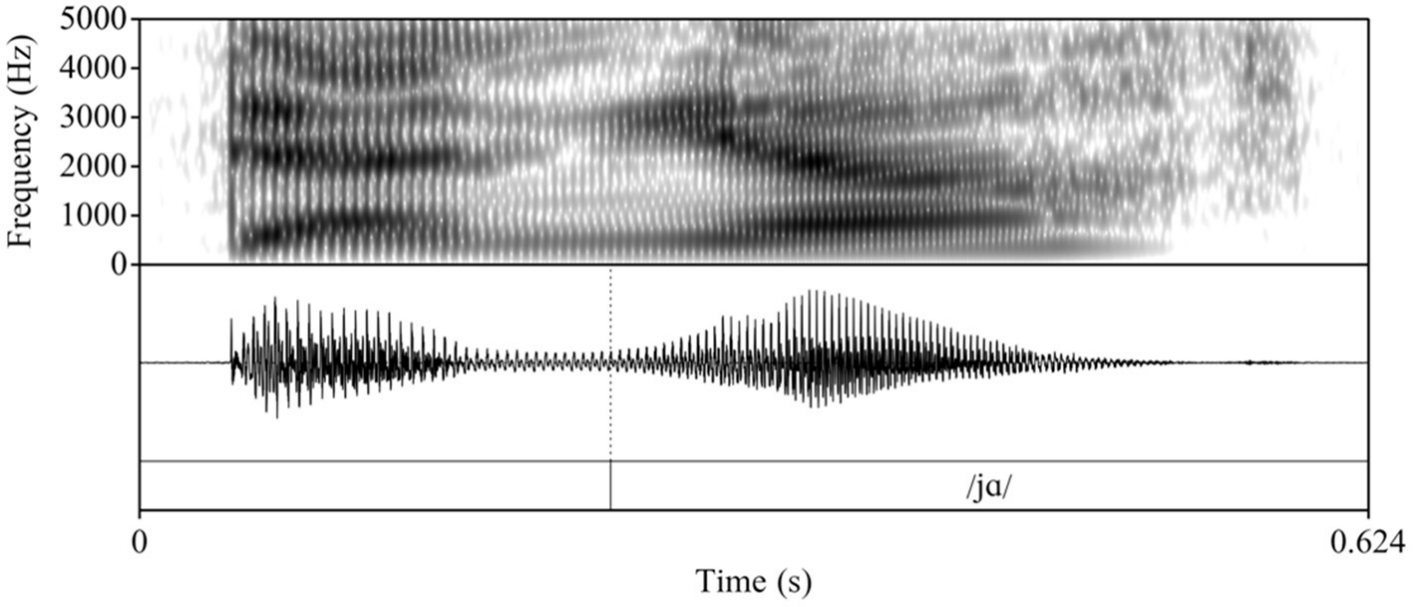
Waveform and spectrogram of an advanced L2 Spanish participant's production of the English nonce item /dεnja/ “denya.”

**Figure 2:**
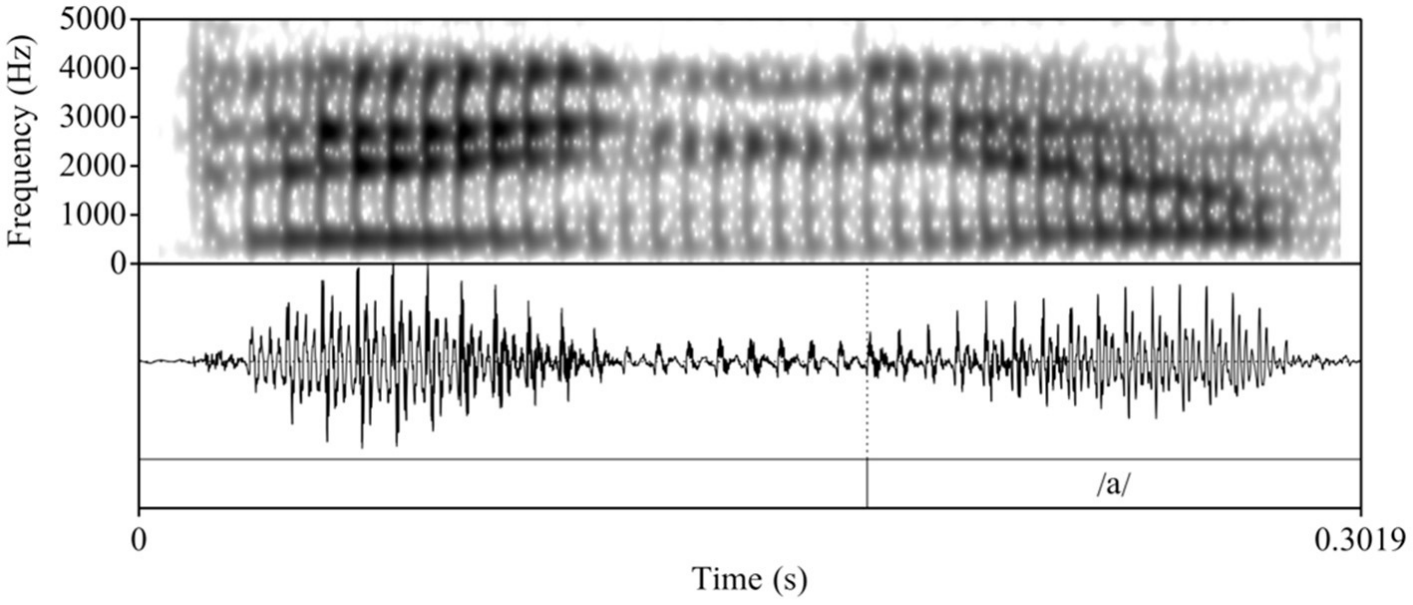
Waveform and spectrogram of an L1 Spanish participant's production of the Spanish nonce item /deɲa/ “deña.”

**Figure 3:**
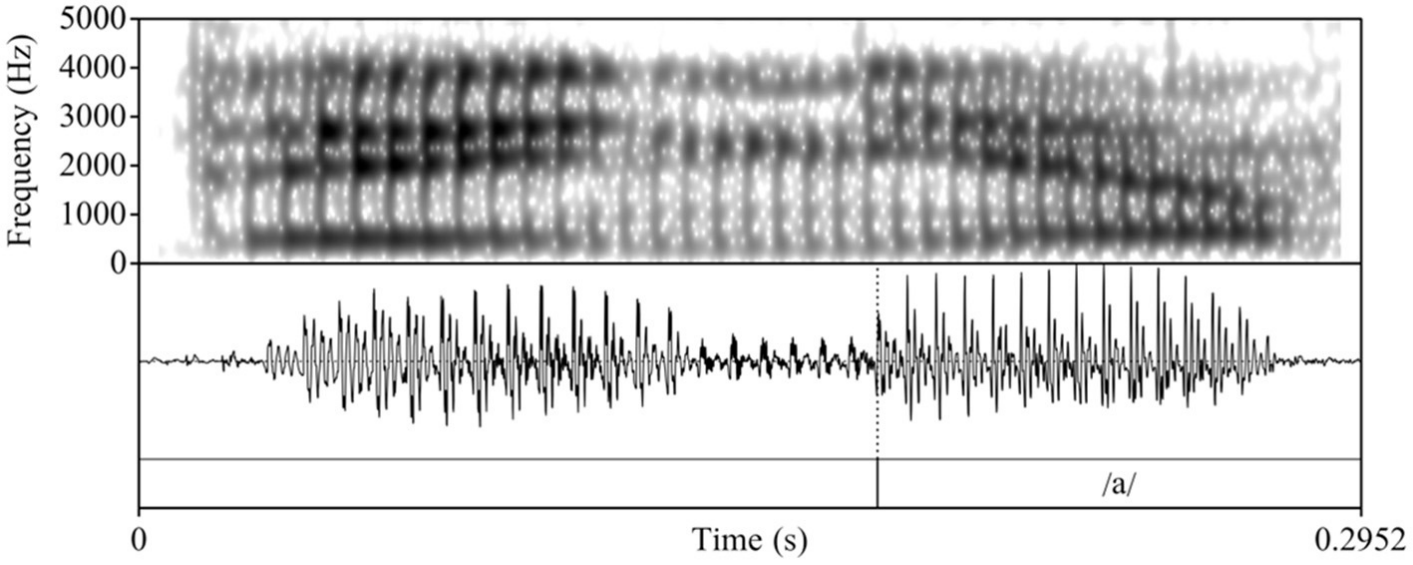
Waveform and spectrogram of an L1 Spanish participant's production of the Spanish nonce item /dena/ “dena.”

To determine whether L2 Spanish learners produce a single segment (/ɲ/) or a sequence (nj), we follow Bongiovanni (2019) by acoustically examining the vocalic portion that follows the nasal segment^4^. Specifically, we measure duration and first and second formant (F1 and F2) contours as correlates of the phonological association of a glide element. In her comparison of the production of /nj/^5^ and /ɲ/ in Buenos Aires Spanish, Bongiovanni examined reported differences in gestural timing, specifically, sequential and quasi-simultaneous alveolar and palatal contact in /nj/ and /ɲ/, respectively (see e.g., Recasens and Romero, 1997). While some speakers evidenced neutralization of /nj/ and /ɲ/, the speakers who preserved the contrast exhibited formant contour trajectories that differed in the first (F1) and second (F2) formants, with /nj/ showing a rise in F2 and lowering of F1 and a later F1 minimum and F2 maximum compared to /ɲ/. Duration of the vocalic portion in /nj/ was longer than in /ɲ/, given the glide's independent status. To our knowledge, there are no crosslinguistic comparisons of Spanish /ɲ/ and English /nj/^6^. Therefore, we rely on the tautosyllabic Spanish data to form the logical prediction that the distinction between heterosyllabic English /nj/ and Spanish /ɲ/ will be qualitatively similar to Spanish /nj/ vs. /ɲ/ and potentially more pronounced given the association of English /j/ with the onset of the following syllable. Figures 1–3 illustrate the differences in formant trajectory and duration of the following vocalic portion by an advanced L2 Spanish participant of English /nj/ (Figure 1) compared with an L1 Spanish /ɲ/ (Figure 2) and L1 Spanish /n/ (Figure 3), the latter of which we include as a baseline for comparison with /nj/ and /ɲ/.

### L2 Acquisition of Complex Palatal(ized) Consonants

As mentioned, although this is the first study to our knowledge to examine L1 English speakers' acquisition of the L2 Spanish palatal nasal, we can look to a small body of research that has examined L1 English speakers' acquisition of L2 Russian palatalized consonants to inform predictions for the current study. Similarly to Spanish /ɲ/, and unlike the English /nj/ sequence, Russian palatalized consonants are single complex segments with dual places of articulation that contrast phonemically with non-palatalized counterparts. Therefore, the L2 learning task for L1 English speakers is similar. In onset position^7^, it seems as though L2 learners are able to perceive Russian /C^j^/~/C/ contrasts (Larson-Hall, 2004; Kulikov, 2011). However, it is not clear from these studies whether learners accurately perceive the distinction as /C^j^/~/C/ or whether the operation of L1 constraints instead persists in driving perception of the contrast as /Cj/~/C/. Diehm (1998) and Lukyanchenko and Gor (2011) examined this question as it relates to perception of the /C^j^/ ~ /C^j^j/ contrast (akin to the Spanish /ɲ/ ~ English /nj/ distinction), with Diehm also reporting production data of within-language and between-language contrasts.

Regarding perception of the /C^j^/ ~ /C^j^j/ contrast, Lukyanchenko and Gor's data from an ABX task reflected above-chance accuracy in low-proficiency (~73%)^8^ and high-proficiency (80%) L2 Russian learners. In an identification task, Diehm found that high-experience learners perceived /C^j^/ and /C^j^j/ more accurately than low-experience learners when followed by a low vowel (23.5 and 10.2% more accurately, respectively) and that the increase in /C^j^/ accuracy corresponded with a decrease in inaccurate identification of /C^j^/ as /Cj/. While the learners in these two studies are neither at ceiling nor in line with L1 Russian accuracy, the data suggest that learners can develop perceptual acuity that at least partially circumvents the L1 phonological constraints that yield /Cj/ perception. Production data from Diehm, the only study to our knowledge to report a within-subjects comparison of L1 English and L2 Russian production data, points to partial acquisition in the oral modality. She found that even advanced L2 Russian learners' productions of /C^j^V/ and /C^j^jV/ syllables did not differ in F2 trajectory or duration from the point of consonantal release to the offset of the palatal element. This result lends support to the observation that L1 English learners decompose L2 /C^j^/ into an L1-like /Cj/ sequence. Interestingly, a crosslinguistic comparison of a subgroup (*n* = 4) of advanced learners' L1 English and L2 Russian productions revealed that, while one learner produced L1 /Cj/ and L2 /C^j^/ as (L1-like) /Cj/, the other three produced a distinct sequence in Russian that fell between their L1 English /Cj/ and the L1 Russian /C^j^/ comparison. That is, although the L2 Russian /C^j^V/ F2 trajectories did not approximate those of the L1 Russian comparison group, they were shorter and had a more negative slope (i.e., a larger degree of gestural overlap) than their English /CjV/. Diehm posited that this intermediate representation was indicative of partial L2 acquisition.

Taking the L2 Russian data as a point of departure, it seems that (at least partially) overcoming the relevant L1 English constraints is possible. However, we recognize that the learning scenario is different in L2 Russian vs. L2 Spanish. Specifically, the functional load (i.e., the importance in marking contrasts in a language) of the Russian /C^j^V/ ~ /CV/ contrast is higher than the functional load of the Spanish /ɲ/~/n/ contrast. Russian, which has 42 consonantal phonemes, has 15 pairs of consonants that are phonemically distinguished by palatalization; palatalized consonant phonemes range in frequency ranking from 14 to 42 (Smirnova and Chistikov, 2011). Recall that Spanish /ɲ/, on the other hand, is the least frequent phoneme in the General Latin American Spanish inventory (Melgar de González, 1976), which contains 17 consonantal phonemes. Moreover, the phoneme is the only palatal consonant with dual articulation in Spanish and only contrasts in its palatalization with /n/. The phoneme thus has low functional load because it is infrequent but occurs in minimal pairs, with a low predictability of distribution. Functional load has been posited as a predictor of L2 phonological acquisition outcomes, whereby the probability of the acquisition of a contrast correlates with the functional load of that contrast (e.g., Best and Tyler, 2007). Relatedly, Archibald (Archibald, 2007, 2009) posits that, for learners to acquire a novel L2 contrast, there need to be sufficiently robust cues in the input to drive a revision to the representation that would allow for accurate perception of a single segment. Thus, it is wholly possible that L2 Spanish learners will not evidence the same success as L2 Russian learners. While the present study was not designed to explicitly test the effect of functional load and cue robustness, we will return to their potential role in the discussion.

### Research Questions and Predictions

There are two research questions that drive the current study. The first regards whether learners acquire the relevant contrast in the L2, independent of how their /ɲ/ productions compare with the L1 Spanish /ɲ/.

(1a) Do L1 English learners of L2 Spanish produce acoustically distinct Spanish /n/ and /ɲ/, as measured by duration and formant trajectories of the following vocalic portion?
(1b) Does the distinction of /n/ and /ɲ/ vary by proficiency?

Following the acoustic description of the nasal segments in section Nasal consonants in English and Spanish, distinct segments are predicted to take the form of a longer vocalic portion following /ɲ/ than following /n/; /ɲ/ is expected to present a higher F2 and a lower F1 than /n/, with an overall flatter shape for /n/ vs. /ɲ/.

In the case that learners distinguish /n/ and /ɲ/, the second question concerns the acoustic quality of /ɲ/.

(2a) If learners distinguish /n/ and /ɲ/, (i) do they rely on an L1 representation to produce /ɲ/ or (ii) have they overcome L1 constraints to establish a novel L2 representation? In this latter case, does the acoustic quality of the L2 representation pattern with that of an L1 Spanish representation?
(2b) Does the acoustic quality of the L2 Spanish /ɲ/ vary as a function of proficiency?

There are two logical outcomes. The first is that we will encounter evidence that learners have mapped Spanish /ɲ/ in the input onto their representation of English /nj/. In the Speech Learning Model (SLM, Flege, 1995, 2002; Flege and Bohn, 2020), this process of “equivalence classification” is predicted to eventually yield a representation (in SLM terms, a “phonetic category”) that subsumes English /nj/ and Spanish /ɲ/ and has shifted to accommodate properties of both sounds. If one or both groups' /nj/ and /ɲ/ pattern together, we can compare their /nj/ and /ɲ/ productions with an L1 English baseline and an L1 Spanish baseline to determine where the learners might be in the equivalence classification process. The second possible outcome is that the learners have overcome the relevant L1 constraints and acquired a novel representation of Spanish /ɲ/ that is distinct from their L1 English /nj/. In this case, the difference is predicted to take the form of (a) shorter duration of Spanish /ɲ/ than English /nj/ and/or (b) a formant contour wherein /nj/ has a lower F1 valley and higher F2 peak than /ɲ/.

Based on Diehm's (1998) L2 Russian production data discussed in section L2 acquisition of complex palatal(ized) consonants, we can make tentative predictions with the caveat that the L2 Spanish developmental trajectory may diverge from that of the L2 Russian trajectory due to the status of the palatalized segments in Spanish vs. Russian. We predict that learners will distinguish /n/ from /ɲ/ in production even at beginner proficiency (RQ 1). They will approximate /ɲ/ in earlier stages of acquisition via L1-like /nj/; in later stages the duration and formant trajectory of /nj/ in L2 Spanish will shift toward the L2 target but will not fall within the acoustic parameters of the L1 comparison group's productions (RQ 2), resulting in an intermediate representation.

## Methods and Materials

### Participants

Twenty-seven Spanish/English bilinguals participated in this study. Participants were all undergraduate or graduate students at a Midwest University at the time of testing ranging in age from 19 to 42 (*M* = 25.80, *SD* = 5.24). The Spanish/English bilinguals were divided into three groups based on order of acquisition and level of proficiency: (1) L2 Beginner (*n* = 9), 2) L2 Advanced (*n* = 8) and (3) L1 Spanish (*n* = 10). The L2 Beginner and L2 Advanced groups are comprised of L1 English speakers who learned Spanish as an L2. The L1 Spanish baseline comparison group mirror the L2 groups and are L1 Spanish speakers who learned English as an L2. The use of a Spanish baseline from a mirror-image bilingual group avoids the problematic comparison of bilinguals to monolinguals and acknowledges that a bilingual's systems do not act in isolation (see e.g., Grosjean, 2010). Further, as noted by an anonymous reviewer, the use of this baseline group is appropriate in the context of L2 learners in the United States, as it is often the case that learners' interactions are largely with bilingual Spanish speakers including, but not limited to, their instructors.

As measures of L2 language proficiency, L2 Spanish participants completed a 50-item multiple-choice test consisting of portions of the Diploma of Spanish as a Foreign Language (DELE) and Modern Language Association (MLA) that was first used in Slabakova and Montrul (Slabakova and Montrul, 2003) and has been widely used in L2 Spanish research; L1 Spanish participants completed a 50-item English proficiency cloze test adapted from the Oxford Placement Test. The L1 Spanish participants' English proficiency mirrors that of the L2 Advanced group's Spanish proficiency. Further, participants also completed the Bilingual Language Profile, BLP (Birdsong et al., 2012) as a proxy for language dominance. The BLP is a biolinguistic questionnaire that asks questions about bilinguals' language use, language acquisition, etc. and calculates a score for language dominance on a scale of−218 (Spanish dominant) to 218 (English dominant) with “0” indicating “balance” between the two languages. Further, as a part of the BLP, participants rate their Spanish and English proficiency with respect to reading, writing, speaking and understanding. Table 1 illustrates the participant demographics by group.

**Table 1:** Participant information.

		**L2 Spanish Beginner**		**L2 Spanish Advanced**		**L1 Spanish**	
		***M***	***SD***	***M***	***SD***	***M***	***SD***
Age of Spanish acquisition		14.00	3.20	14.50	4.21	Since birth	
Age of English acquisition		Since birth		Since birth		9.67	4.21
BLP dominance score		145.21	33.67	91.73	15.91	−75.99	25.59
Spanish proficiency score (out of 50)		20.33	5.09	45.38	2.92	n/a	
English proficiency score (out of 50)		n/a		n/a		43.50	2.87
**Spanish self-rated proficiency**							
	Reading	2.44	1.33	5.50	0.53	6	0
	Understanding	2.33	1.00	5.25	0.70	6	0
	Speaking	1.56	0.88	5.13	0.83	6	0
	Writing	1.89	1.36	5.25	0.46	6	0
**English self-rated proficiency**							
	Reading	5.89	0.33	6	0	5.56	0.52
	Understanding	5.89	0.33	6	0	5.33	0.50
	Speaking	5.89	0.33	6	0	4.67	0.50
	Writing	5.78	0.67	6	0	4.56	0.53

### Materials

The experiment consisted of Delayed Repetitions Tasks (e.g., Trofimovich and Baker, 2006) in English and Spanish. There were 40 trials (10 critical, 10 control, 20 distractor) in each task. A trial consisted of a target disyllabic nonce word with penultimate stress embedded within a carrier phrase, i.e., *Digo X para ti* in Spanish and its equivalent “I'm saying X to you” in English. A 1,000 ms pause followed the carrier phrase, after which participants were prompted to repeat the original sentence with the question *¿‘Qué me dices?* In Spanish and its equivalent “What are you saying to me?” in English. All items were phonotactically licit in the target language: Critical and control items in Spanish consisted of (C)CV1.ɲV2 and (C)CV1.nV2 structures, respectively; critical and control items in English consisted of (C)CV1n.jV2 and (C)CV1.nV2 structures, respectively. Across conditions, V1 was a mid or low vowel (/ε/ or /ɲ/ in English; /e/ or /o/ in Spanish) and V2 was /a/ in Spanish and /ɲ/ in English. Distractors followed the same general (C)CV.CV structure as the control and critical stimuli. English and Spanish stimuli were recorded by phonetically trained female native speakers of Midwest American English and Northern Peninsular Spanish, respectively. Table 2 illustrates the item composition for the English and Spanish tasks.

**Table 2:** Spanish and English stimuli.

	***n***	**English**	**Example**		**Spanish**	**Example**	
Critical	10	(C)CVn.ja	/dεnja/ ['dεn.jə]	“denya”	(C)CV.ɲa	/deɲa/ ['de.ɲa]	*deña*
Control	10	(C)CV.na	/dεna/ ['dε.nə]	“denna”	(C)CV.na	/dena/ ['de.na]	*dena*
Distractor	20	(C)CV.CV	/lεka/ ['lε.kə]	“lecka”	(C)CV.CV	/meba/ ['me.β_T_a]	*meba*

Trials were presented using E-prime (Psychology Tools, Inc.); audio stimuli were presented over Sennheiser HD-280 PRO headphones through a MOTU Ultralite mk3 interface. Recordings were made in a sound-attenuated booth using a head-mounted Shure SM 10A dynamic microphone and a Marantz PMD 661 solid-state recorder at a 44.1 kHz sampling rate.

### Procedure

The experiment was conducted in a single session divided into Spanish mode and English mode segments, with the language mode order counterbalanced across participants. After providing informed consent, participants started the first segment with a 10-min interview in order to establish the first language mode, followed by the delayed repetition task in that language. The English mode segment ended with completion of the BLP and the Spanish mode segment ended with the Spanish written proficiency assessment. The L1 Spanish comparison group only participated in the Spanish mode segment and completed the interview, repetition task, and English written proficiency assessment, in that order.

### Analysis

#### Duration Analysis

##### Acoustic Analysis

Following the research questions presented in section Research questions and predictions, this study examines the duration and formant trajectories of the following vocalic portion as acoustic indices to differentiate between nasal segments. To that end, we used Praat [6.1.16] (Boersma and Weenink, 2020) to segment and analyze the sound files. The theoretical ceiling of tokens was 710 or 30 per L2 learner (10 Spanish critical, 10 English critical, 10 Spanish control) + 20 per L1 control (10 Spanish critical, 10 Spanish control). Eighteen tokens were removed from data analysis due to non-target productions (participants repeating, skipping or producing different segments), creaky voice, or background noise for a final total of 692 tokens.

**Figure 4:**
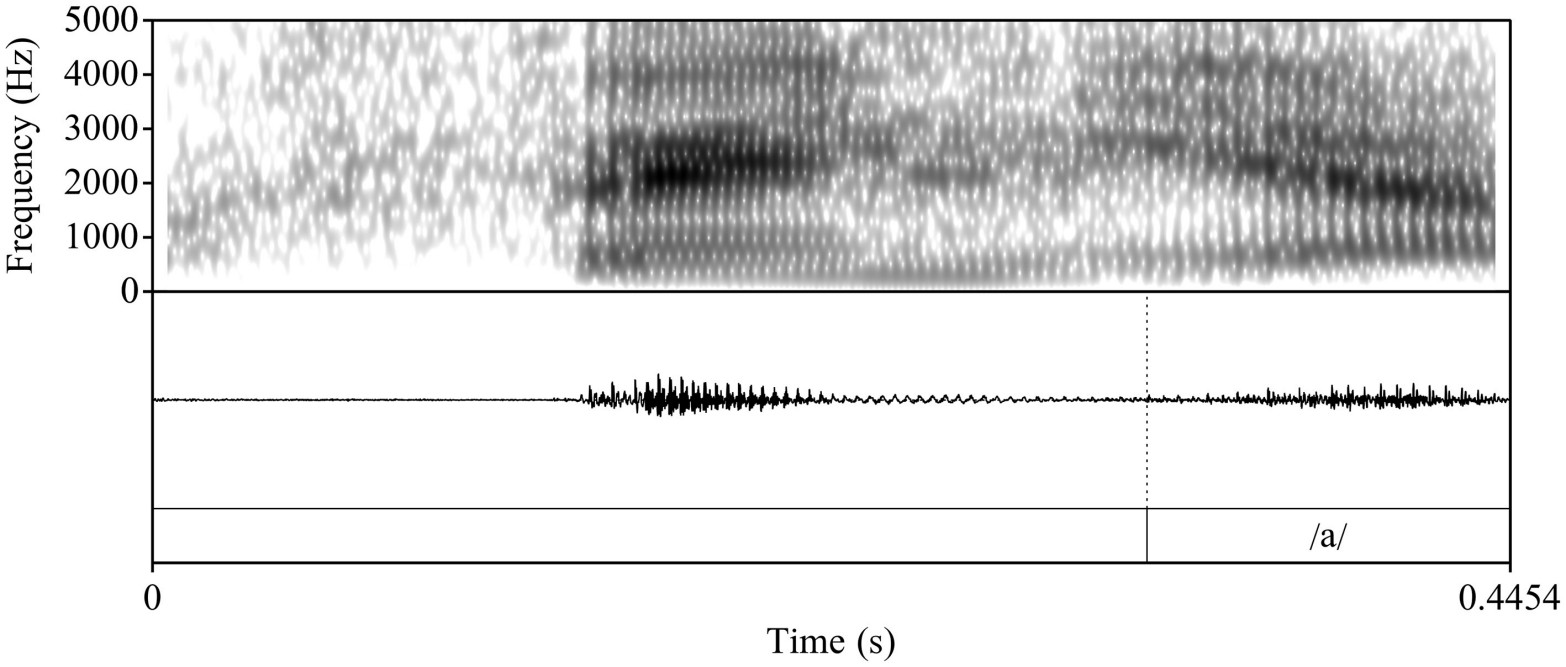
Waveform and spectrogram of a L2 Spanish participant's target-like production of /ñ/ the Spanish nonce item /feña/ “feña.”

**Figure 5:**
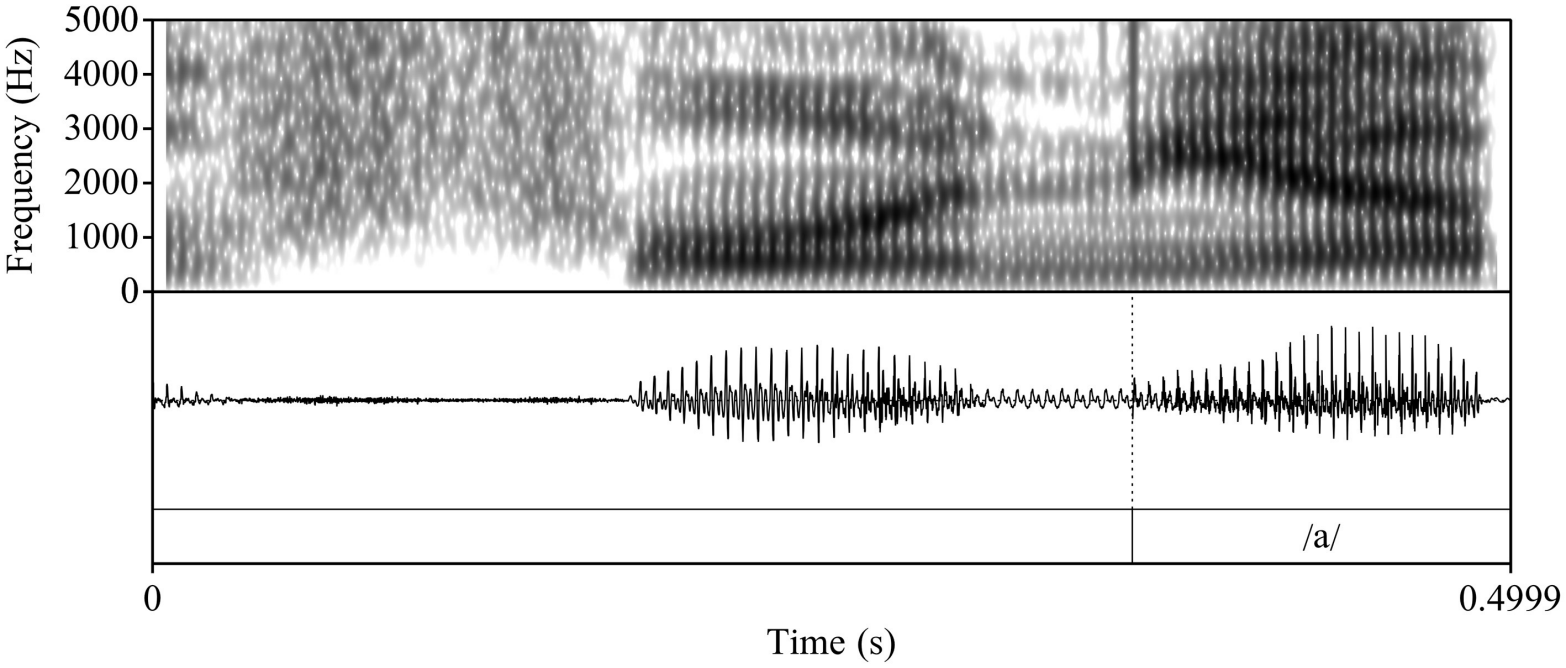
Waveform and spectrogram of an L2 Spanish participant's L1 English-like production of /ñ/ in the Spanish nonce item /feña/ “feña.”

During segmentation, we used the following cues to determine the onset and offset of the vocalic portion: 1) the visual presence of an abrupt change in formant structure and frequencies (onset) and 2) a breaking up of the formant structure and a loss of energy and periodicity in the waveform (offset). Following Bongiovanni (Bongiovanni, 2019), boundaries between formant transitions or between the glide and the vowel /a/ were not marked. Figures 4, 5 illustrate two L2 productions of Spanish /ɲ/ and their segmentation: Figure 4 aligns with the L1 /ɲ/ in Figure 2, in which /ɲ/ is represented by a steeper transition (i.e., slope) from the offset of the nasal into the following vowel /a/ (when compared with that of /nj/). Figure 5, on the other hand, aligns more closely with the L1 English /nj/ in Figure 1, in which the formant transition between the nasal segment and following vowel is marked by a raise in F2 frequency and a decrease in F1. After segmentation, we analyzed the sound files by using Praat scripts to extract the measurements (Hirst, 2012, for automatic duration measurements; McCloy and McGrath (2012), for semi-automatic formant measurements). Formant measurements were taken at 20 points within the vocalic portion (i.e., every 5%).

##### Statistical Analysis

For duration of the vocalic portion, in order to normalize for potential between-participants differences in speech rate, we transformed raw duration to z-scores for each participant, with separate transformations for the L2 participants in English and Spanish. While English z-scores were transformed on /nj/ items and /n/ items, only /nj/ items were included in the analysis since the English /n/ data are not relevant to our research questions. In consideration of the sample size, rather than fitting the data to linear mixed-effects models, we follow Plonsky (Plonsky, 2015, p. 30) and instead rely on a combination of 95% confidence intervals (CIs) and effect sizes (here, Hedges' g, which corrects for bias from small sample size) to evaluate between-subjects and within-subjects differences. When using CIs for between-participants comparisons, a difference in means is significant when one group's mean does not fall within the comparison group's CI (Plonsky, 2015, p. 40). For within-groups comparisons, two means are considered significantly different if the CI of the mean of the two differences does not cross zero (Cumming and Finch, 2005). Small, medium, and large effect size thresholds are based on Plonsky and Oswald (Plonsky and Oswald, 2014), whereby between-participants thresholds are.40,0.60, and 1.00, and within-groups thresholds are 0.60, 1.00, and 1.40, respectively.

For the formant trajectories of the vocalic portion, we followed the analysis carried out in Bongiovanni (Bongiovanni, 2019). We transformed the formant values to Bark units and a Smoothing Spline ANOVA (SSANOVA) was fit to the data (time points and corresponding Bark units at each time point) in R, version 4.0.2 (R Core Team, 2020) with the gss package. As part of this analysis, a smoothing spline fits a smooth curve to the data and the SSANOVA determines whether the curves in question are statistically different from one another. Statistical significance is measured by non-overlapping confidence intervals around the splines. Following previous research (e.g., Simonet et al., 2008; Nance, 2014; Kirkham, 2017; Bongiovanni, 2019), we report only the graphical representations of the SSANOVA.

## Results

### Research Question 1

#### Duration

**Figure 6:**
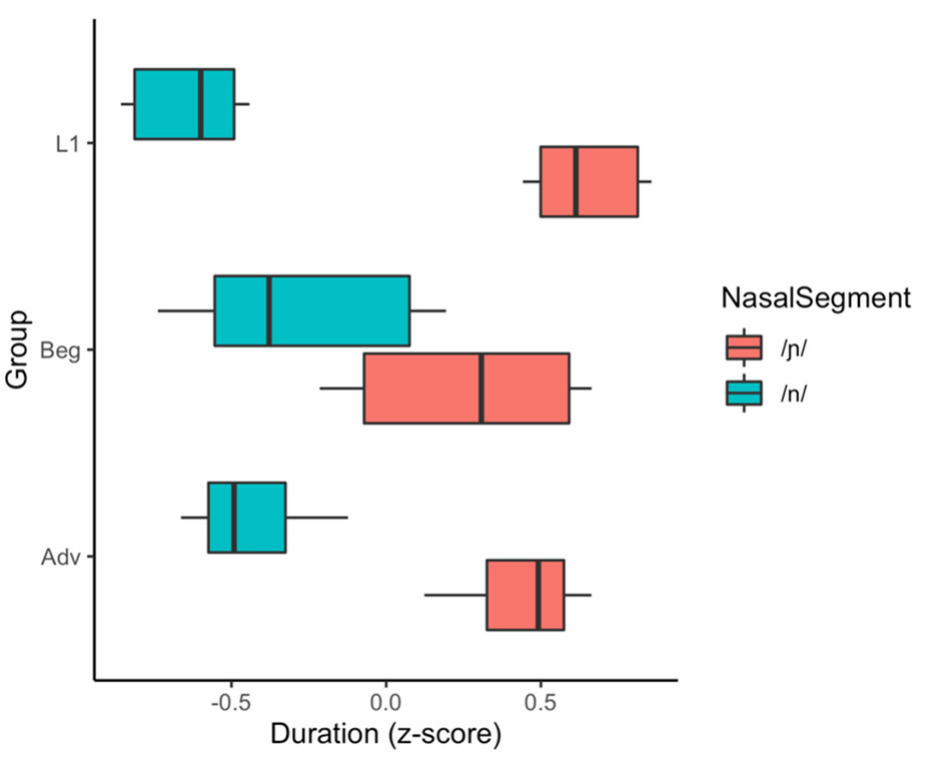
Z-score transformed duration of Spanish /n/ and /ɲ/ produced by the beginner and advanced L2 groups and L1 Spanish group.

To determine whether learners produce distinct /n/ and /ɲ/ as measured by duration and whether durational differences are moderated by proficiency, the analysis included within-subjects comparisons of the learners' English and Spanish productions as well as between-groups comparisons of the beginner vs. advanced learners. As shown in Table 3 and Figure 6, the durational difference for /n/ and /ɲ/—whereby the vocalic portion following /ɲ/ would be predicted to be longer than that following /n/—was not significant for the beginner group and the effect size was negligible, with a large CI indicative of substantial variation within the group. However, the difference was significant for the advanced group with a medium effect size. All between-group comparisons were significant: The advanced group falls between the beginner group and L1 Spanish group, who make an even larger durational distinction between /n/ and /ɲ/.

**Table 3:** RQ 1: within-groups durational difference between /n/ and /ɲ/ for each group.

	**Beg**				**Adv**				**L1**			
	**M**	**SD**	**CI^a^**	**g**	**M**	**SD**	**CI^a^**	**g**	**M**	**SD**	**CI^a^**	**g**
/n/	−0.26	0.99	[−1.40, 0.37]	0.52	−0.46	0.87	[−1.73, −0.11]^c^	1.02^b^	−0.64	0.72	[−1.98, −0.59]^c^	1.67^b^
/ɲ/	0.25	0.89			0.46	0.86			0.65	0.75		

*g, Hedges' g*.

a *CI of the difference between the two means*.

b *Hedges' g > 0.6*.

c *CI of the difference does not cross zero*.

#### Formant Trajectories

**Figure 7:**
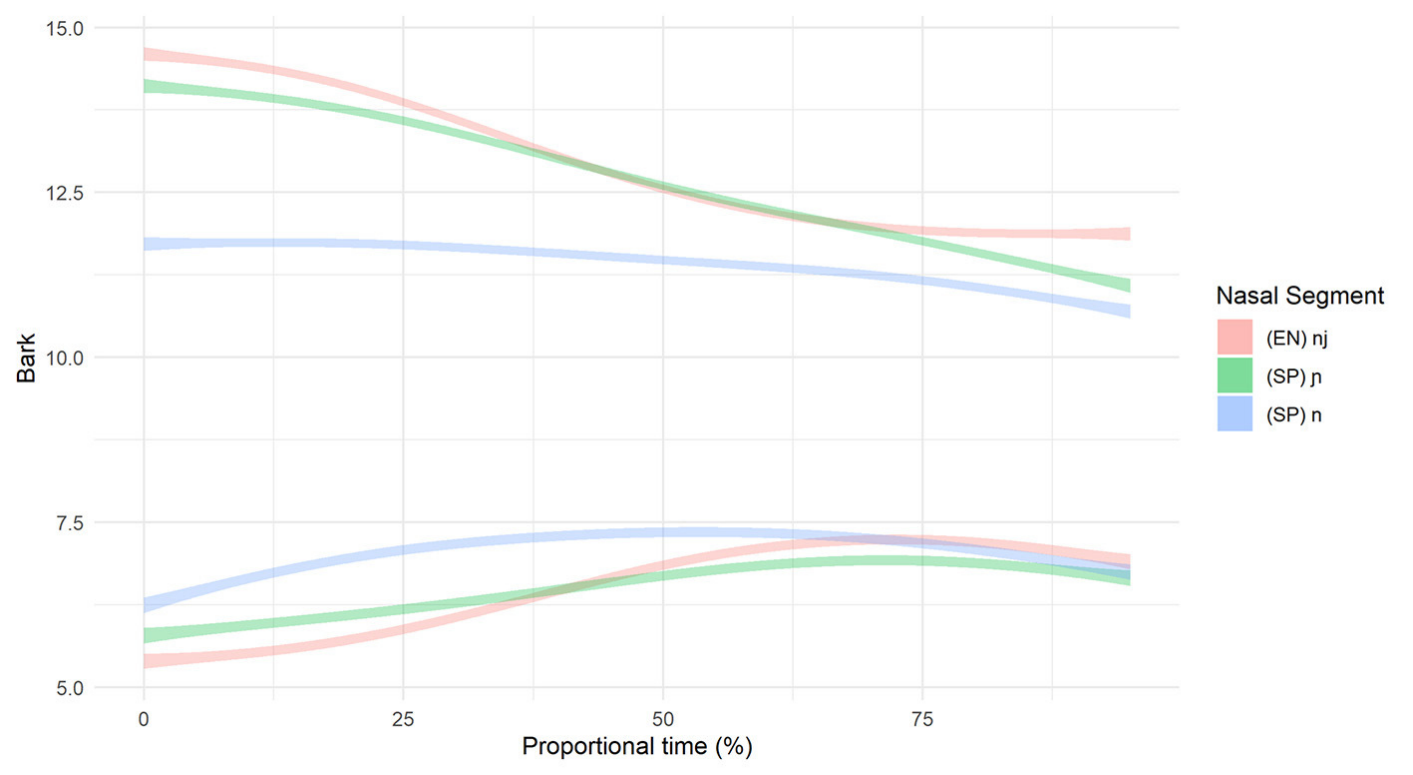
Smoothing Spline ANOVA of formant trajectories for the L2 beginner group.

**Figure 8:**
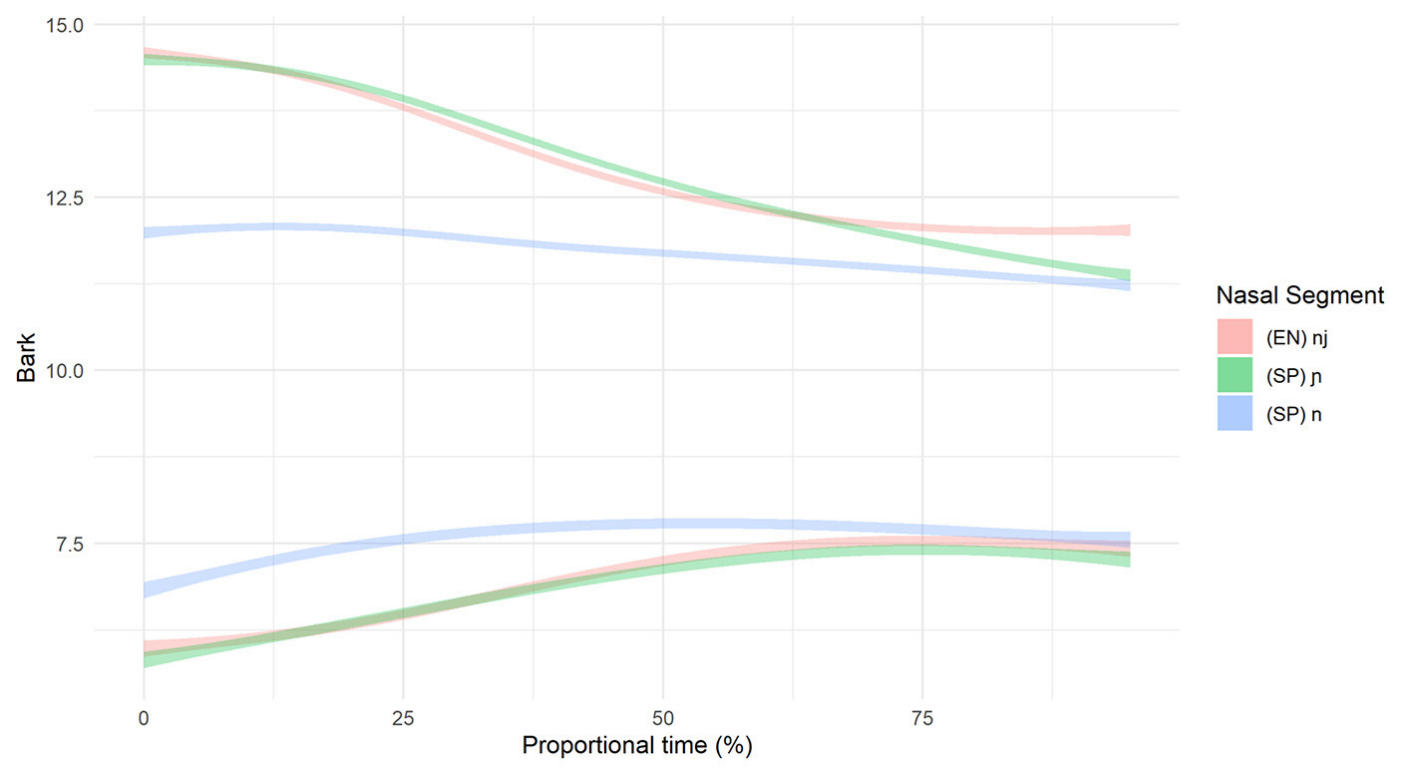
Smoothing Spline ANOVA of formant trajectories for the L2 advanced group.

For the differences in formant structure, recall that with SSANOVA, statistical significance is indicated by non-overlapping confidence intervals plotted around the data-generated formant curves. With that in mind, Figures 7, 8 present the results of the SSANOVA for the beginner (Figure 7) and advanced (Figure 8) L2 groups' productions of English /nj/, Spanish /ɲ/, and Spanish /n/.

For the beginner L2 group, the formant trajectories are marked by non-overlapping confidence intervals. There is zero overlap comparing F1 in /n/ and /ɲ/ and no overlap between 0 and 85% of the F2 curve, with maximum differences of 0.94 and 2.41 Bark units, respectively. Similar results present for the advanced L2 group: There is zero overlap in the confidence intervals for both F1 and F2 when comparing /n/ and /ɲ/, with a maximum difference of 1.24 Bark units and 2.52 Bark units for F1 and F2, respectively.

To summarize the results for RQ 1: While the beginner group showed no significant difference between /n/ and /ɲ/ in terms of duration, there was a significant difference in the formant trajectories. The advanced group showed a significant difference between /n/ and /ɲ/ for both duration and formant trajectories.

### Research Question 2

#### Duration

**Figure 9:**
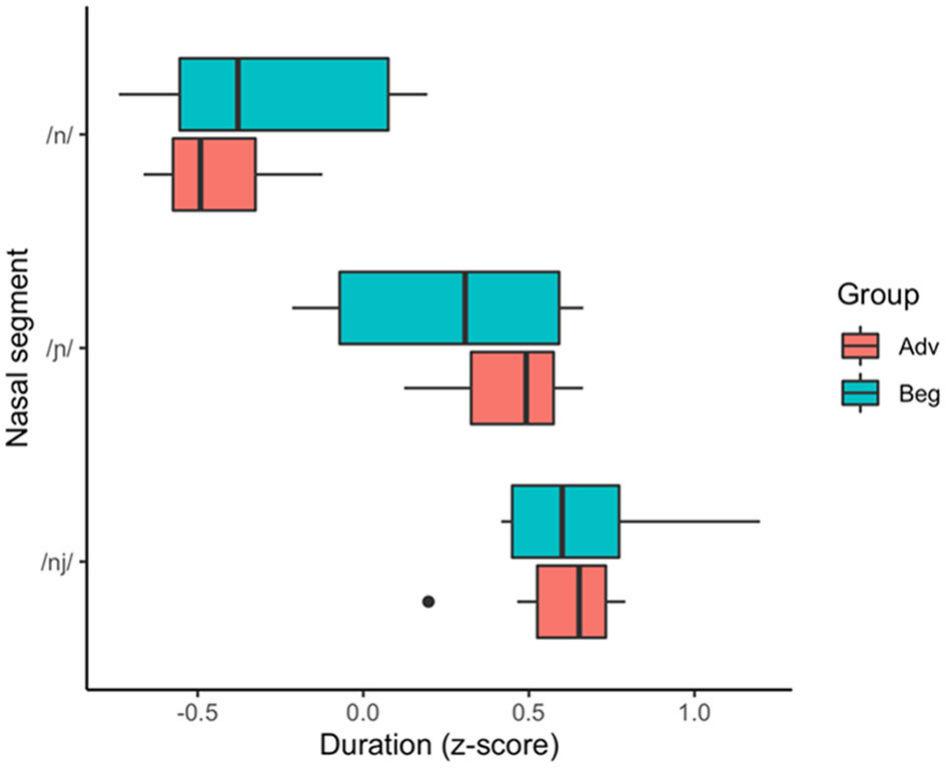
Z-score transformed duration of Spanish /n/, Spanish /ɲ/, and English /nj/ produced by the beginner and advanced L2 groups.

Because the beginner group does not produce durationally distinct /n/ and /ɲ/ and the advanced group does, we limit our duration analysis as it relates to RQ 2 to the advanced data. We examined whether the acoustic quality of the advanced group's duration of /ɲ/ reflects (i) perceptual mapping of /ɲ/ to English /nj/ or (ii) development of a novel L2 representation. We first compared the advanced learners' /ɲ/ to their English /nj/ and to the L1 English baseline (i.e., the beginner group's English /nj/. Figure 9 provides a visual indication of the proximity of the advanced learners' /ɲ/ to their /nj/ as well as the proximity of the advanced /nj/ to the L1 English baseline (beginner) /nj/. The visual trends are supported by the data in Table 4; the advanced group did not make a significant durational distinction and the effect size did not reach the minimum threshold for a small effect. Moreover, a between-group comparison of the beginner and advanced /nj/ (Table 5) shows no difference.

**Table 4:** RQ 2a: advanced learners' within-groups comparison of English /nj/ and /ɲ/.

	**Adv**			
	**M**	**SD**	**CI^a^**	**g**
/ɲ/	0.46	0.86	[-0.94,0.58]	0.21
/nj/	0.64	0.76		

*g, Hedges' g*.

a *CI of the difference between the two means*.

**Table 5:** RQ 2b: beginner (L1 English baseline) and advanced learners' between-groups comparison of English /nj/.

	**/nj/**			
	**M**	**SD**	**CI**	**g**
Beg	0.61	0.86	[0.43, 0.79]	0.04
Adv	0.64	0.76	[0.47, 0.81]	

*g, Hedges' g*.

Interestingly, Figure 6 also shows the advanced learners' /ɲ/ trending with the L1 /ɲ/, and while the data in Table 6 show that the advanced mean falls outside the L1 CI, the L1 CI falls on the edge of the advanced CI and the effect size does not approach the minimum threshold for a small effect. We also see in the same table that the L1 /ɲ/ is not different than the advanced /nj/.

**Table 6:** Comparison of L1 group /ɲ/ with advanced group /ɲ/ and /nj/.

	**Adv /ɲ/**				**Adv /nj/**			
	**M**	**SD**	**CI**	**g**	**M**	**SD**	**CI**	**g**
Adv	0.46^b^	0.86	[0.27,0.65]	0.22	0.64	0.76	[0.47,0.81]	0.00
L1 /ɲ/	0.65	0.75	[0.50,0.80]		0.65	0.75	[0.50,0.80]	

*g, Hedges' g*.

b *Mean does not fall within comparison group's CI*.

#### Formant Trajectories

**Figure 10:**
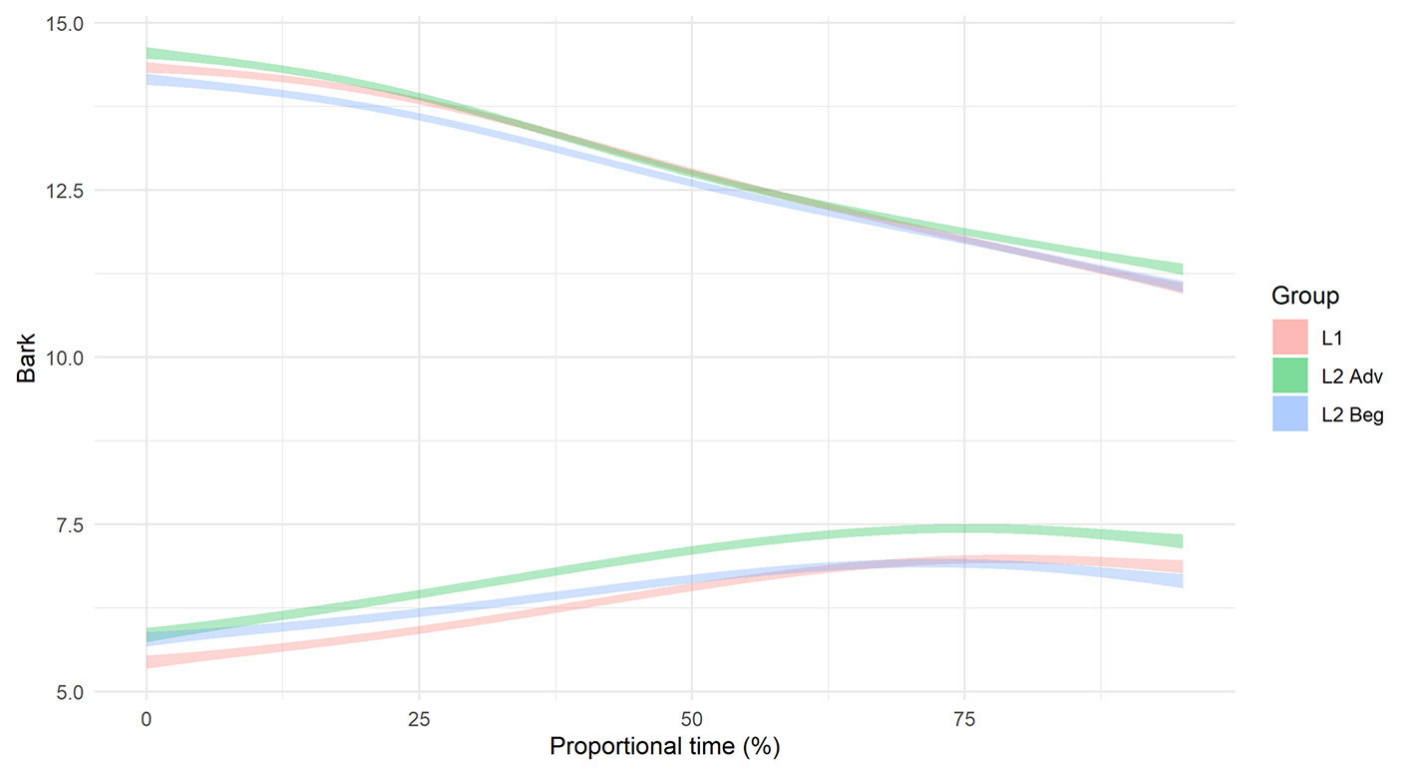
Smoothing Spline ANOVA formant trajectories of Spanish /ɲ/ by group.

To inform the nature of the L2 groups' production of Spanish /ɲ/, the formant structures were subject to a within-groups comparison (L2 Spanish /ɲ/ vs. L1 English /nj/) and a between-groups comparison (L2 Spanish /ɲ/ vs. L1 Spanish /ɲ/). Recall that, in addition to non-overlapping confidence intervals, differences between English /nj/ and Spanish /ɲ/ are expected to present in the form of a higher F2 peak and lower F1 valley for English /nj/ vs. Spanish /ɲ/. Keeping that in mind, Figures 7, 8 illustrate the within-groups comparisons for Beginner and Advanced L2 groups, respectively, and Figure 10 illustrates the between-groups comparison.

For the Beginner L2 group, the within-group comparison (Beginner /ɲ/ vs. Beginner /nj/) revealed no overlap in confidence intervals for F1 (maximum difference =0.47 Bark), with the exception of the point where the two curves cross at 35–45%. The same pattern presents for F2, with no overlap in confidence intervals between 0–35 and 70–100% (maximum difference =0.51 Bark) and an overlap between 35 and 70% where the formant curves cross. The formant trajectories reflect the expected differences between /nj/ and /ɲ/, i.e., a lower F1 valley and a higher F2 peak for /nj/ vs. /ɲ/. The between-groups comparison (Beginner /ɲ/ vs. L1 Spanish /ɲ/) revealed no overlap in F1 confidence intervals between 0–55% and 85–100% (maximum difference =0.32 Bark), with an overlap between 55 and 85% where the curves cross. Further, there was no overlap in F2 confidence intervals between 0 and 65%, with a maximum difference of 0.51 Bark.

For the advanced L2 group, the within-groups comparison (Advanced /ɲ/ vs. Advanced /nj/) revealed that the F1 confidence intervals overlap between 0 and 50% and then run adjacent to one another from 50 to 100%. The F2 confidence intervals overlap at the beginning of the formant trajectory (0–20%) and then again when the curves cross around 65%. There is no overlap between 20 and 55% nor between 70 and 100% (maximum difference =0.21 Bark). Visually, the formant trajectories illustrate a higher F2 peak for English /nj/ vs. Spanish /ɲ/ but a lower F1 valley for Spanish /ɲ/ vs. English /nj/. However, the confidence intervals are overlapping at both of these points, thus rendering this distinction non-significant. The between-groups comparison (Advanced /ɲ/ vs. L1 Spanish /ɲ/) revealed zero overlap in the F1 confidence intervals with a difference of 0.64 Bark units at their most different. The F2 confidence intervals do not overlap at the beginning and the end of the trajectory (between 0 and 20% and between 75 and 100%) with a maximum difference of 0.22 Bark units). Visually, the Advanced /ɲ/ demonstrates a higher F2 peak (but not a lower F1 valley) when compared with the L1 control /ɲ/.

To summarize the results for RQ 2: As with RQ1, there were no differences in duration within learner groups or between learner groups and the L1 Spanish group. In terms of the beginners' formant trajectories, there was a significant three-way distinction between the beginners' Spanish /ɲ/, their English /nj/, and the L1 Spanish /ɲ/. In contrast, the advanced data's considerable overlap between /nj/ and /ɲ/ formant contours suggests a lack of difference. However, comparably limited overlap between the advanced /ɲ/ and L1 /ɲ/ contours indicate a significant difference in formant trajectories (see section structural equation modeling for details).

## Discussion

This study examined the speech production of beginner and advanced groups of L1 English/L2 Spanish learners and a comparison group of L1 Spanish/L2 English speakers to determine whether their production evidences a phonemic distinction between Spanish /n/ and /ɲ/, and, if so, whether learners establish the contrast via L1 English /nj/ or creation of a novel L2 representation. Between-segment patterns were established via two acoustic indices: Z-score transformed durations of the vocalic portion that follows the nasal segment and formant trajectories of the same vocalic portion. Durational differences were predicted to take the form of longer duration for /nj/ than /ɲ/ and for /ɲ/ than /n/. The formant trajectory for /nj/ was expected to consist of an F1 with a lower valley and an F2 with a higher peak compared with /ɲ/; /n/was predicted to evidence an earlier and higher F1 peak and an overall lower F2 contour.

Before we turn to the discussion of the results, there are two notes regarding our analysis: First, because this is the first study to measure these sounds in L1 English/L2 Spanish bilinguals and there are no data to inform relative cue strength for these contrasts, we avoid the arbitrary assignment of relative weights to the indices of duration, vowel height (F1), and vowel frontedness (F2). Instead, we treat them as three separate strategies that speakers may use to distinguish between these nasal segments. Second, we eschew an arbitrary quantification of how little overlap in the confidence intervals of the spline curves constitutes a meaningful difference between the nasal segments and focus our qualitative interpretation on the first half of the formant trajectories (0–50%). In doing so, we home in on the nature of the transitions in /nj/ vs. /ɲ/ rather than differences in the vocalic portion, which is expected to differ due to cross-linguistic differences in the following vowel ([ə] in English vs. [a] in Spanish).

### Research Question 1

Our first research question asked whether L1 English learners' L2 Spanish production reflects a distinction between /n/ and /ɲ/ in Spanish and if that distinction is subject to differences in proficiency. Beginning with the duration data, our beginner group did not evidence a significant difference in duration between /n/ and /ɲ/ but the advanced group did, producing longer vocalic portions following /ɲ/ than /n/ with a medium effect size. This difference between the learner groups suggests that the durational difference increases as a function of L2 proficiency. In comparison to the advanced group, however, the L1 Spanish group distinguishes via duration to a greater degree. Thus, while the advanced L2 Spanish and L1 Spanish both distinguish the segments via duration, the degree to which the L2 group does so does not approximate the L1 Spanish comparison.

If we were to use duration as the only acoustic index of the /n/~/ɲ/ distinction, we would conclude that beginner learners have not acquired the /ɲ/ phoneme in Spanish since there is no durational difference between Spanish /n/ and /ɲ/. However, the formant trajectory data indicate that the beginners and advanced learners alike utilize height (F1) and frontedness (F2) of the vocalic portion to distinguish /n/ and /ɲ/. Figures 7, 8 illustrate majority non-overlap between the F1 and F2 formant contours for both beginners and advanced learners. In other words, in response to RQ1, the data indicate that yes, both groups of learners produce acoustically distinct Spanish /n/ and /ɲ/ segments. Further, the distinction varies by proficiency, with the beginners relying largely on F1 and F2 structure and the advanced learners utilizing both F1 and F2 structure and duration.

**Figure 11:**
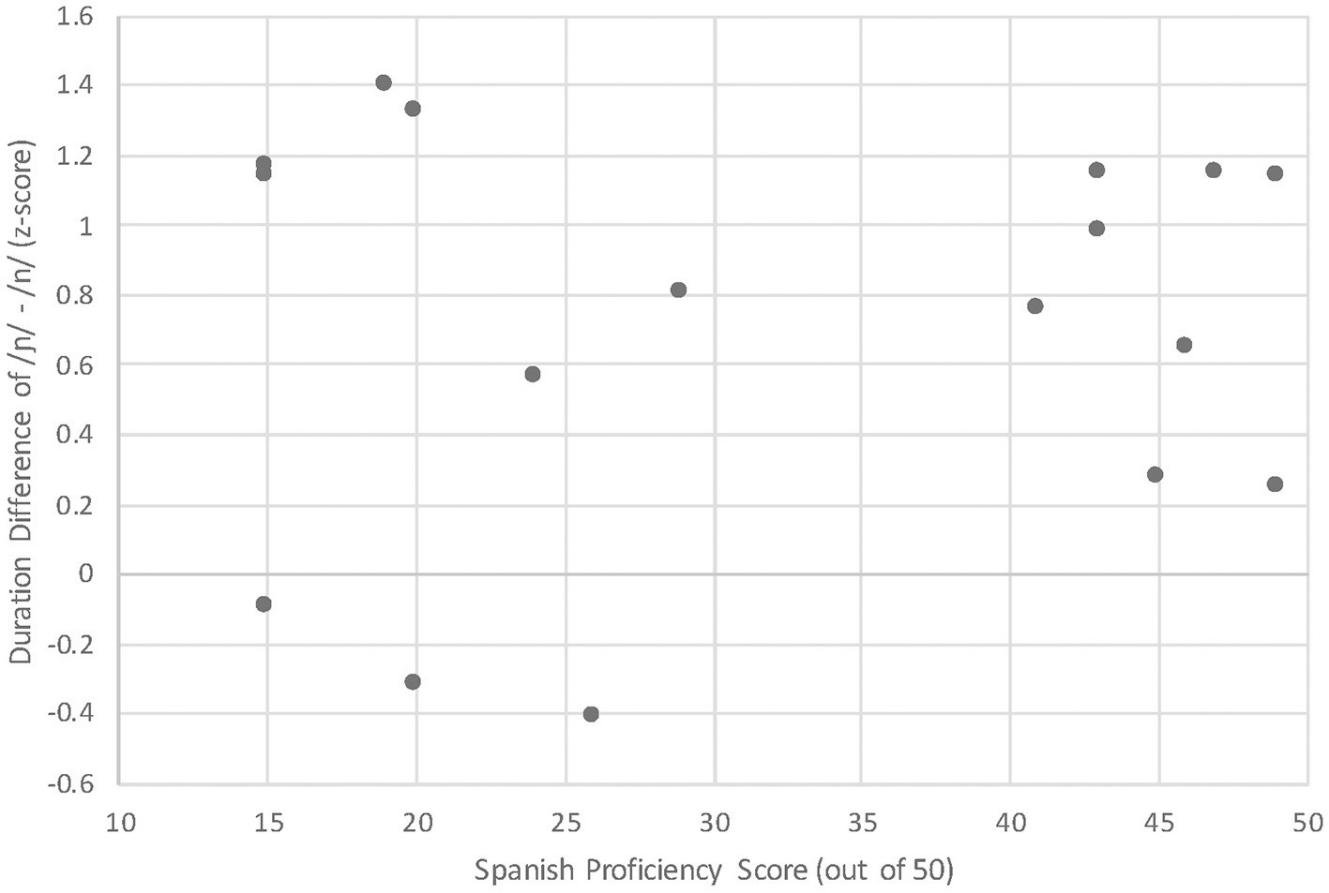
Learners' duration difference in the following vocalic portion of /ɲ/ – /n/ by Spanish proficiency score.

Given that the Spanish /n/~/ɲ/ contrast varies by proficiency, the question that follows is: Why do beginner learners use vowel height and frontedness to make a distinction, but not duration? There are two points to consider. First, it could be the case that duration is not the primary cue that learners attend to in the input to distinguish the /n/~/ɲ/ contrast, but rather that it is a later-acquired cue that learners have available to them at advanced proficiencies (see e.g., Kong and Lee, for discussion of the effects of proficiency on L2 cue-weighting strategies). Second, we remind the reader of the large standard deviation values for the duration results (Table 3), which indicate substantial variation within each proficiency group. For a more nuanced understanding of the relationship between proficiency and the use of duration, we plotted each learner's durational difference by their Spanish proficiency score (Figure 11).

While the group data indicated that the use of duration to differentiate /n/ and /ɲ/ was restricted to advanced proficiency, the individual data plotted by proficiency score indicate a range of durational difference across scores without a discernable pattern. That is, we do not see a clear relationship between an increase in proficiency score and an increase in duration difference to maximize the distance between /n/ and /ɲ/. This visualization is bolstered by a very weak positive correlation [*r*_(17)_ = 0.08, *p* = 0.760]. Thus, the individual data are suggestive of individual differences in cue weighting, which have been documented in L2 acquisition (e.g., Chandrasekaran et al., 2010; Clayards, 2018), and specifically in the use of duration vs. formants in vowel discrimination (Kim et al., 2018). To confirm this hypothesis, we will need data from the perception of stimuli that isolate the acoustic indices and their possible combinations. Perception data from L1 and L2 Spanish speakers will inform the relative cue strength used by early vs. late learners of Spanish and longitudinal examination will inform whether L2 cue-weighting strategies change as a function of proficiency, as reported in Kong and Lee (2018). In addition, L1 Spanish perception of the L2 Spanish production data will be necessary to confirm that the quantitative differences in duration and formant contours are meaningful (in this case, perceivable).

### Research Question 2

**Figure 12:**
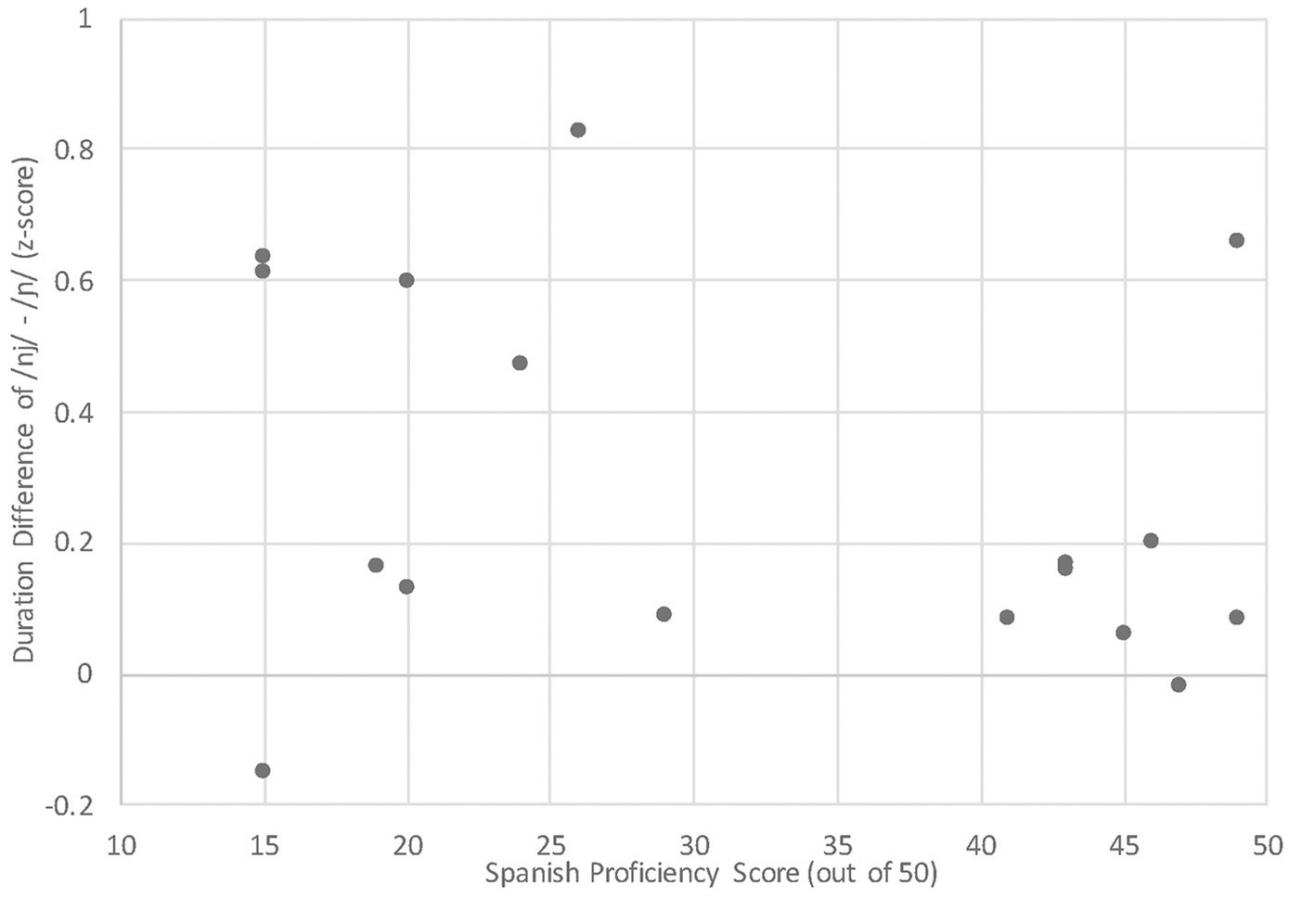
FV Duration Difference [nj] - [ɲ] by Spanish proficiency score.

Our second research question concerned the quality of the learners' Spanish /ɲ/. That is, (a) do they rely on their L1 English /nj/ to approximate the novel Spanish contrast, or (b) have they overcome L1 constraints and established a single segment? We begin with the duration results, which are limited here to the advanced group since the beginner group did not use duration to contrast Spanish /n/ vs. /ɲ/. Since the learners' Spanish /ɲ/ was not different from their English /nj/, we posit that they do not use duration to differentiate them. Solely based on this outcome, we might conclude that the learners rely on English /nj/ to approximate the L2 Spanish /ɲ/ target. However, neither of these was different from the L1 Spanish /ɲ/. What might explain a scenario in which a learner's L1 and L2 sounds do not differ from each other and also do not differ from the L2 target? One possibility is that the learners' L2 Spanish /ɲ/ has affected their L1 English /nj/. L2 influence on the L1 aligns with a scenario of equivalence classification in which /ɲ/ is initially mapped onto /nj/ and, over time, the representation shifts in the direction of the L2 sound. Nevertheless, the advanced learners' English /nj/ did not differ from the English baseline (i.e., the beginner English /nj/ data). These inconclusive findings cast doubt on the reliability of duration as an acoustic correlate in this case, at least at the group level. A look at the individual-level duration difference between the advanced learners' English /nj/ and Spanish /ɲ/ (Figure 12, proficiency scores 40–50) supports the group data, with all but one advanced participant's differences clustered around zero. While there was a weak negative correlation between proficiency score and duration difference [*r*_(17)_ = −0.30, *p* =0.237], the weakness is likely due to the variation in the beginners' duration differences (proficiency scores < 30).

Turning to the formant data, we first compare the L2 learners' English /nj/ and Spanish /ɲ/, followed by the L1 Spanish and L2 Spanish /ɲ/. The beginners use vocalic quality to distinguish between English /nj/ and Spanish /ɲ/: They differentiate via vowel height (F1) and frontedness (F2) as illustrated by non-overlapping formant contours in the first half of the vocalic portion that follows the nasal segment (see Figures 7, 8). The advanced learners, however, do not differentiate via F1, and the F2 contours overlap at the critical onset. Comparison of Spanish /ɲ/ across the three groups (Beginner, Advanced, L1 Spanish) shows a clear difference between the Beginner /ɲ/ and L1 Spanish /ɲ/ via F1 and F2, with no overlap in the critical regions. The Advanced /ɲ/ and L1 Spanish /ɲ/ comparison, however, is less straightforward. For F1, there is no overlap, although the shape of the formant contour is similar; for F2, there is no overlap at the onset.

Based on these comparisons of duration and formant contours, our tentative response to RQ 2a is that (i) the advanced group relies on their L1 /nj/ representation when producing Spanish /ɲ/ while the beginner group does not, and (ii) neither group approximates the L2 Spanish target as measured by an L1 Spanish baseline^9^. In the case of the beginner group, they appear to have established an intermediate representation, although, as we note in our discussion of RQ 1, we will need perception data to determine whether the attested quantitative differences are perceivable. Considering that all of the maximum differences fell below the JND threshold of 1 Bark unit, these data are particularly warranted. Regarding RQ 2b, the acoustic realization of Spanish /ɲ/ varies as a function of proficiency: The beginners and advanced realizations differ from each other according to height and frontedness. Both groups differ from the L1 Spanish baseline along both parameters, although the advanced group approximates the L1 more closely than the beginner group.

**Figure 13:**
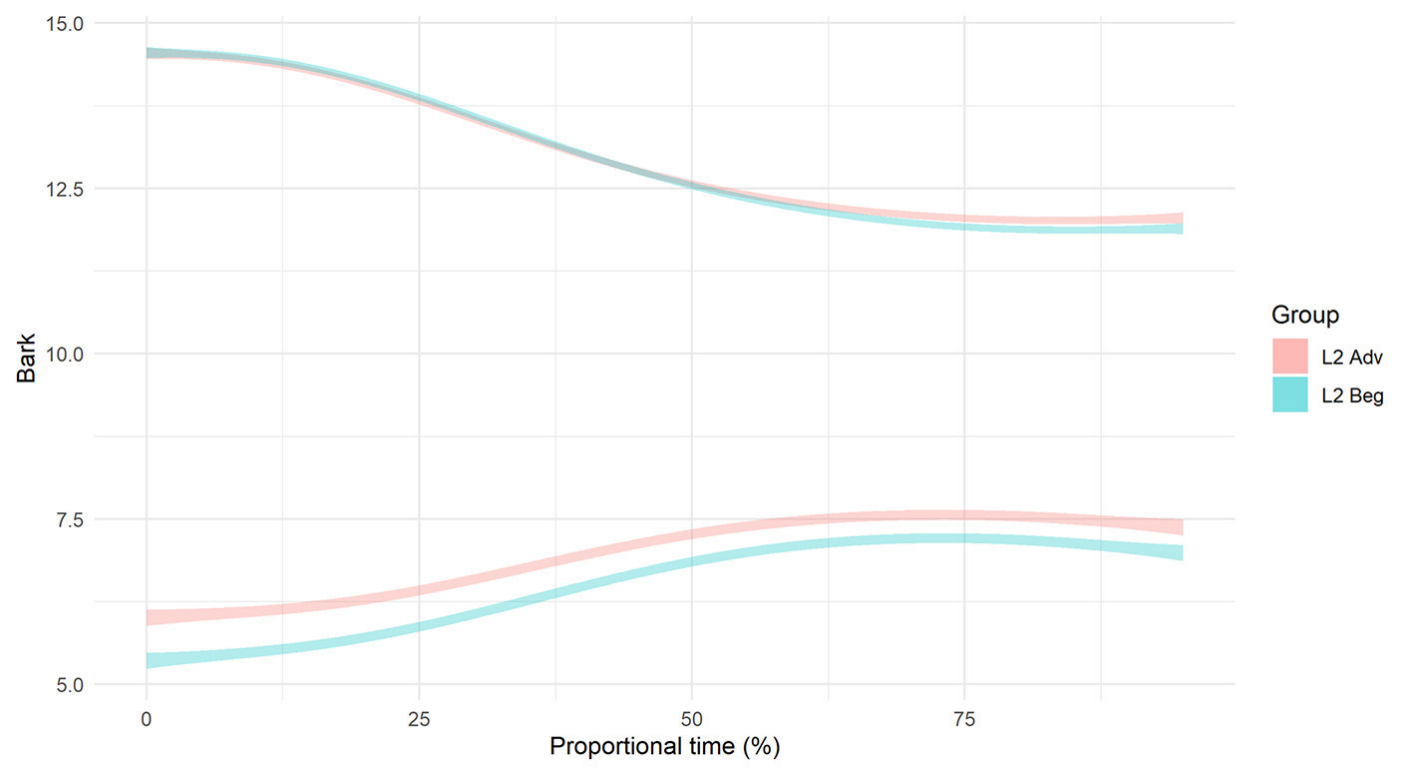
Smoothing Spline ANOVA of English /nj/ formant trajectories.

The finding that beginner and advanced L2 learners differ from one another as well as from the L1 Spanish baseline is not unexpected; intermediate representations have been commonly documented in L2 production research (Zampini, 2008 for a review; see e.g., Broselow and Kang, 2013). In fact, recall that this is what Diehm (Diehm, 1998) found when comparing advanced L2 Russian learners' productions of palatalized consonants (section L2 acquisition of complex palatal(ized) consonants). The unexpected result, however, is that the advanced learners' productions (and not the beginners') show a persistent L1 effect. A common L2 developmental trajectory consists of initial pervasive L1 influence on the L2. Over time, these effects are thought to lessen as the L2 grammar develops, eventually yielding an L2 representation that (often partially) converges on the L2 target. This attested pattern has been formalized in models such as Major's (2001) Ontogeny Phylogeny Model (OPM), which explains the relationship between transfer, universals, and similarity. Of particular relevance to the present case is the OPM's Similarity Corollary, which posits that L1 transfer effects are persistent in later stages of development when the L1 and L2 phenomena are similar. These effects are thought to limit the role of universals, access to which is necessary to overcome L1 constraints, slowing down the L2 acquisition process. While the advanced learners produce the relevant L1 and L2 sounds similarly and thus align with this pattern, consideration of the beginner and advanced data in tandem suggest a case of U-shaped learning that can be likened to phonological regression attested in child phonological development (see e.g., Tessier, 2019). That is, it is possible that learners initially establish a novel (albeit intermediate) representation. Later, they recognize that an established L1 representation can be redeployed in the L2 (and potentially without compromised intelligibility, see discussion in section Results), which triggers the mechanism of equivalence classification. As noted in section L2 acquisition of complex palatal(ized) consonants, the shared representation is predicted to eventually shift to accommodate properties of both sounds. As we did with duration above, we can gauge the potential shift of /nj/ by comparing the Advanced /nj/ with the L1 English baseline (here, Beginner /nj/) (Figure 13).

What we find is that the F1 contour is indeed different, but in the opposite direction of what would be predicted (i.e., a higher – rather than lower–F1 valley), and there is no difference in F2. Thus, there is no evidence of a shift toward Spanish. Going forward, longitudinal data will best inform the following outstanding questions regarding the relationship between /ɲ/ and /nj/ via within-group developmental observations: (1) Do learners who first establish a three-way crosslinguistic distinction (/n/~/ɲ/~/nj/) eventually develop a single representation that is used for perception of English /nj/ and Spanish /ɲ/? (2) In the case of a shared representation, does the representation trend in the direction of the L1 or L2? And finally, as a complement to the variables we have examined here: (3) Is the representation that learners use to produce /ɲ/ tautosyllabic or heterosyllabic? That is, is there evidence that learners can overcome the L1 English constraint that militates against /nj/ in onset position? To address this question, future analyses will (a) examine production data for syllabic breaks via identification of glottalization (Scarpace and Mirza, 2014; González and Weissglass, 2017; Scarpace, 2017) and/or pauses (González and Weissglass) and measurement of preceding vowel duration (Scarpace and Mirza, 2014) and (b) test the perception of /nj/ in onset position vs. heterosyllabic position.

## General Discussion and Conclusion

In this study, we have seen that learners are able to produce a novel L2 contrast even at a lower level of proficiency, but that the quality of the L2 representation does not approximate the L2 target. The logical question that follows then, is why not, particularly in the case of the advanced learners? In the remainder of the discussion, we consider two factors first introduced in section L2 acquisition of complex palatal(ized) consonants that have been shown to influence outcomes in L2 phonology—the functional load of a contrast (e.g., Best and Tyler, 2007) and the robustness of acoustic cues (e.g., Archibald, 2007, 2009)—and consider the practical implications of the attested outcomes.

First, recall Diehm's (1998) finding that highly proficient L1 English/L2 Russian speakers established a novel /C^j^/ representation that differed from their English /Cj/ representation, thus outperforming the advanced L2 learners in our study. We could reasonably hypothesize that the difference between the L2 Russian and L2 Spanish learners can be attributed to the comparatively higher functional load of the contrast in Russian. However, the L2 Russian learners' new representation did not converge on the L2 target, which suggests that high functional load might be necessary but not sufficient for L2 convergence.

Instead, a lack of L2 convergence might be at least partially attributed to cue robustness. Archibald (2007, 2009) presented the hypothesis that acoustic cues must be sufficiently robust to trigger changes in the grammar that would yield accurate perception. The perceptual strength of the relevant cues in this case might be insufficient for an L2 learner; in fact, they might be insufficient even for L1 Spanish speakers: Bongiovanni (2015) reported that L1 Spanish speakers in Buenos Aires did not distinguish /ɲ/ and /nj/^10^ in perception, even though a recent study shows that this same speaker population did so in production (Bongiovanni, 2019). If an L1 speaker cannot reliably perceive a difference, it is reasonable to predict that an L2 speaker cannot either, which could (at least partially) explain fossilization of a compromise or intermediate representation. That is, a shift in representation, while not “native-like,” might never be triggered since intelligibility, or “the extent to which a listener understands a speaker's message” (Munro and Derwing, 2006), is not compromised. Another possibility, however, is that the learners have in fact established a target-like phonological representation that is simply not reflected in production. Following the approach of direct mapping from acoustics to phonology (DMAP, Darcy et al., 2012), a learner may establish an L2 phonological contrast prior to acquisition of a target phonetic representation. This is because the formation of lexical contrasts “do[es] not require attunement to target-like category boundaries”; rather, construction of the relevant feature matrices “requires only the detection of acoustic correlates of phonological features in the raw percepts” (p. 16)^11^. Triangulation of our production data with perception data that reflect both categorization and discrimination will provide further insight into the learners' developmental trajectories.

Returning to the question of intelligibility, if an L2 speaker's message is not at risk of being lost, what are the practical implications of these findings? Pronunciation pedagogy objectives have shifted away from adherence to native-speaker norms and toward intelligibility (see e.g., Levis, 2018, for discussion). With this shift in mind, if intelligibility is not compromised, we posit that the limited instructional time that teachers have to dedicate to pronunciation does not need to be spent on this contrast. In fact, if /ɲ/ is addressed in Spanish pedagogical materials, it typically uses the English heterosyllabic /nj/ as a teaching tool, with statements such as “In speech, this letter [ < ñ>] sounds like the middle sound in “canyon” and, in fact, the Spanish word for “canyon” is *cañon*” (Diversity Style Guide, 2020). This type of information could actually reinforce an intermediate /nj/ representation via conversion of explicit knowledge to implicit knowledge (see e.g., Ellis, 2015 for discussion of this relationship). As a complement the longitudinal observation of perception and production we have proposed, debriefing data on learners' experience with explicit instruction will help elucidate the effects of formal instruction on fossilization.

## Data Availability Statement

The raw data supporting the conclusions of this article will be made available by the authors, without undue reservation.

## Ethics Statement

The studies involving human participants were reviewed and approved by University of Illinois Office for Protection of Research Subjects. The patients/participants provided their written informed consent to participate in this study.

## Author Contributions

SS and JC contributed equally toward conceptualization, methodology, and validation of the project, as well as preparation of the manuscript. SS was responsible for data collection and data analysis. JC was responsible for the theoretical framework. All authors contributed to the article and approved the submitted version.

## Conflict of Interest

The authors declare that the research was conducted in the absence of any commercial or financial relationships that could be construed as a potential conflict of interest.

## Footnotes

^1^To our knowledge, there is only a single study of L2 Spanish /ɲ/ (Díaz-Campos, 2004), which relies on impressionistic data. Although the author reports target-like production of the segment, it is not clear whether the target criteria differentiated between a single segment versus a two-segment sequence. That is, it is possible that the learners were producing heterosyllabic /nj/ rather than /ɲ/.

^2^While the sound appears word-initially, the limited inventory of lexical items is largely composed of loans from indigenous languages or onomatopoeia.

^3^We distinguish the variety of English spoken by the learners in the current study from varieties of English that permit /nj/ in onset position in words such as ‘nuclear’.

^4^In line with Bongiovanni (2019), we avoid acoustic analysis of the nasal segment due to its reported unreliability (see, e.g., Fujimura, 1962, cited in Bongiovanni, 2019, p. 4).

^5^For ease of exposition, we follow Bongiovanni's (2019) phonemic notation of /nj/ and acknowledge that doing so conflates phonological and phonetic representations since the glide is not phonemic in Spanish.

^6^As far as we are aware, the only comparison of English /Cj/ with a language that has a complex palatal segment comes from Diehm (1998); her examination of F2 trajectories found that L1 English /C_[labial]_j/ formant transitions at consonantal release were longer and contained a shallower slope than L2 Russian /C^j^/.

^7^Other studies such as Hacking (2011) and Hacking et al. (2016) have also examined L2 Russian palatalized consonants, but focus on coda position, where, as Hacking et al. (2016) notes, /Cj/ decomposition is not a possible compensatory strategy.

^8^Accuracy rates were presented graphically in a bar chart without numeric labels; exact percentages are not available.

^9^Recalling that our L1 baseline are bilingual Spanish/English speakers, it is possible that a comparison of our baseline group to Spanish monolinguals could reveal differences in /ɲ/ production. However, we are limited in the current study to Spanish data from the baseline and future research will need to include their English data to examine the potential effect of L2 English /nj/ on L1 Spanish /ɲ/.

^10^/ɲ/and /nj/ are contrastive in Spanish, although they form very few minimal pairs (e.g., *huraño* /uȷaɲo/ ‘unsociable’ and *uranio* /uranjo/ ‘uranium.’

^11^See Baker (2004) for an overview of feature geometry analyses of Spanish palatals.
